# Review of Bacterial Nanocellulose-Based Electrochemical Biosensors: Functionalization, Challenges, and Future Perspectives

**DOI:** 10.3390/bios13010142

**Published:** 2023-01-14

**Authors:** Samuel Chagas de Assis, Daniella Lury Morgado, Desiree Tamara Scheidt, Samara Silva de Souza, Marco Roberto Cavallari, Oswaldo Hideo Ando Junior, Emanuel Carrilho

**Affiliations:** 1 Grupo de Pesquisa em Energia e Sustentabilidade Energética-GPEnSE, Universidade Federal da Integração Latino-Americana—UNILA, Av. Sílvio Américo Sasdelli, 1842, Foz do Iguaçu 85866-000, PR, Brazil; 2 Instituto de Química de São Carlos, Universidade de São Paulo, São Carlos 13566-590, SP, Brazil; 3 Instituto Nacional de Ciência e Tecnologia de Bioanalítica-INCTBio, Campinas 13083-970, SP, Brazil; 4 Departamento de Engenharia de Bioprocessos e Biotecnologia, Universidade Tecnológica Federal do Paraná—UTFPR, Campus Dois Vizinhos, Dois Vizinhos 85660-000, PR, Brazil; 5 School of Electrical and Computer Engineering, University of Campinas (Unicamp), Av. Albert Einstein 400, Campinas 13083-852, SP, Brazil; 6 Academic Unit of Cabo de Santo Agostinho (UACSA), Universidade Federal Rural de Pernambuco (UFRPE), Rua Cento e Sessenta e Três, 300-Cohab, Cabo de Santo Agostinho 54518-430, PE, Brazil

**Keywords:** bacterial nanocellulose, biosensors, ex situ, functionalization, in situ

## Abstract

Electrochemical biosensing devices are known for their simple operational procedures, low fabrication cost, and suitable real-time detection. Despite these advantages, they have shown some limitations in the immobilization of biochemicals. The development of alternative materials to overcome these drawbacks has attracted significant attention. Nanocellulose-based materials have revealed valuable features due to their capacity for the immobilization of biomolecules, structural flexibility, and biocompatibility. Bacterial nanocellulose (BNC) has gained a promising role as an alternative to antifouling surfaces. To widen its applicability as a biosensing device, BNC may form part of the supports for the immobilization of specific materials. The possibilities of modification methods and in situ and ex situ functionalization enable new BNC properties. With the new insights into nanoscale studies, we expect that many biosensors currently based on plastic, glass, or paper platforms will rely on renewable platforms, especially BNC ones. Moreover, substrates based on BNC seem to have paved the way for the development of sensing platforms with minimally invasive approaches, such as wearable devices, due to their mechanical flexibility and biocompatibility.

## 1. Introduction

A biomaterial can be defined as “a material designed to interact with living systems and direct the course of any therapeutic or diagnostic procedure” [1]. Biomaterials represent an emergent interdisciplinary research field that has gained attention due to the materials’ advantageous properties, such as nontoxicity, biodegradability, biocompatibility, chemical stability, and having a wide range of applications [2]. The use of biomaterials within the human body has played an increasingly prominent role in the form of implants (heart valves, dental implants, ocular lenses, vascular grafts, etc.) and medical devices (artificial hearts, biosensors, etc.) [3,4]. It is important to note that the features of these biomaterials largely depend on the selection of the material employed for their preparation (e.g., polymer, ceramic, metal, composites, etc.) as well as their particular properties (e.g., material chemistry, material solubility, water absorption, biodegradation, high porosity, and pore size, among others) [5]. From this perspective, natural polymers are one of the categories with the greatest potential for application in biomaterials, especially polysaccharides (e.g., chitosan (CS), cellulose, alginate (Alg), and hyaluronic acid), mainly due to their abundance on earth and superior physicochemical and biological features [6,7,8].

Cellulose is classified as a linear homopolysaccharide composed of β-1,4-linked glucans with a high symmetry level in an amphiphilic molecular structure [9,10]. This carbohydrate and its derivatives are the most abundant biopolymer on the earth and have been extensively researched for biomedical applications in the form of hydrogels, aerogels, films, and fillers due to their remarkable properties and biocompatibility [11,12,13,14,15,16]. Aspects such as the structure and properties of cellulose depend on the source of this polymer, which can be obtained from a wide variety of living organisms, such as plants (e.g., cotton, sisal, wood) [17,18,19] algae (e.g., Valonia) [19], and bacterial sources (e.g., Komagataeibacter) [20]. Among these, the microbial biopolymer produced from bacteria has been one of the most studied celluloses over decades, as it is produced as a nanocellulose network [12,21].

Nanocellulose biosynthesized from bacteria, known as BNC, is a potential alternative source to the other cellulose classes. Its high water-holding capacity [22], high degree of polymerization [23], intrinsic nanostructure [24], high crystallinity [25,26], high mechanical strength [27,28,29], and low cost and sustainable enhanced production [30] contribute to its versatility. Studies have shown that these particular features of BNC, paired with its biocompatibility, make this material an attractive candidate for a wide array of applications (e.g., biomedical, pharmaceutical, biotechnology [31], cosmetics [32], food [8,33], textile [34], and even electronics [35,36,37]). Although pristine BNC lacks conductivity properties compared to other materials [38], this nanomaterial has achieved excellent results as a support for flexible printed electronics [39]. Furthermore, functionalizing nanocellulose with conductive nanoparticles [7] enables it to be used as a nanocomposite-based platform for electronics applications [40], as reinforced conductive papers and films [41], and as bioelectronics devices [40,42].

The concept of bioelectronics [35,43] includes a range of topics at the interface of biology, medicine, and electronics. Due to the studies that have been made on the use of enzymes, antigens/antibodies, or oligonucleotides as a biological interface, the appropriate materials used as support in these devices have received a great deal of attention in the literature [44,45,46,47]. These new biobased materials can guarantee potential applications [35,48] in bioelectronic sensing devices, which have been engineered to provide advances in health care such as clinical diagnosis, detection of pathogens, and other uses [44,49,50,51,52,53].

Biosensors combine a biological recognition layer with a physicochemical transduction layer and an electronic signal processing device that can be employed as an analytical tool to detect an analyte in a wide range of environments [54]. As hybrid devices, they play an essential role by achieving rapid and selective quantitative or semiquantitative analysis when compared to conventional detection methods, such as chromatography and spectroscopy, which are usually costly and time consuming [47,55]. Furthermore, biosensors are low-cost devices that do not require extensive instrumentation and have relatively fast response times due to their chemical redox response [35,51,52,56].

The main challenge in developing these devices has been the often inefficient electron transfer, especially in enzyme-based electrochemical biosensors, between the entrapped enzyme and the surface of the electrode [56]. When it comes to selectivity and affinity, this kind of biosensor can have the wrong operation or a limited ability to function adequately when submitted to real-world samples with complex matrices [56,57]. Intensive studies are now striving to miniaturize the new biosensors and improve biomanufacturing techniques to increase biocompatibility in in vivo monitoring [56]. With this miniaturization in sight, researchers have been making an effort to investigate biomolecule immobilization and electron transport by biomaterial combinations to allow new advances in the bioelectronics field [51]. Furthermore, those working on advances in bioelectronics, and especially in flexible electronics, have taken care to replace unsustainable materials, such as polydimethylsiloxane (PDMS) and polyethylene terephthalate (PET) [58] with those obtained from renewable sources.

BNC-based materials have opened up a wide range of different fields due to their exceptional physical and chemical properties, particularly in biosensing applications [35,59]. Through the integration of BNC with biomolecules and electronic elements, a variety of flexible, biodegradable, and biocompatible platforms with improved electron transfer have been developed [60]. Therefore, this review aims to outline the state of the art in bacterial nanocellulose functionalization techniques and nanocellulose-based composites, presenting the challenges and future prospects of applying BNC in biosensor manufacturing.

## 2. Overall Structure and Preparation of Bacterial Nanocellulose

**Figure 1:**
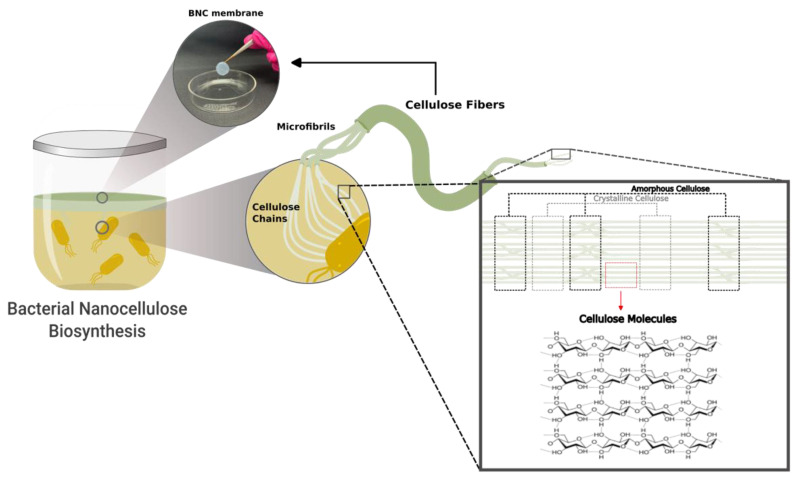
Representation of cellulose structure interconnected by a hydrogen bond. The cellulosic membrane exhibits different structures at different scales: the cellulose fibers consist of bundles of elementary fibrils, and these fibrils are composed of parallel stacked molecular cellulose chains [67].

As a chemical raw material, cellulose ((C_6_H_10_O_5_)n) is the most common polymer in the biosphere [61] and can be considered the most sustainable polymer found on earth, as it is an almost inexhaustible resource [13,62] and possesses a variety of attractive characteristics, such as polyfunctionality and reactivity [63]. This naturally available homopolysaccharide is composed of covalently linked β-1,4-anhydrous-D-glucopyranose units. The hydrogens’ bonds can occur between the hydroxyl groups of cellulose chains (intermolecular hydrogen bonds) or in the same chains (intramolecular hydrogen bonds) [9]. The ability of the hydroxyl groups to form these hydrogen bonds governs the final properties of the cellulose and its derivates [64,65]. The structures of cellulose are represented by two regions: the amorphous domain and the crystalline one. While the first is based on low-ordered cellulose chains, the second contains high-ordered regions. According to these morphological features, the intermolecular hydrogen bonds are the crystalline domains between the hydroxyl groups (Figure 1) [26,66].

The exciting properties of cellulose result from its specific structure, however, its crystallinity, morphology, physicochemistry (density, hydrophilicity, porosity, etc.), and fiber dimensions can vary according to different polymer sources [61]. Thus, its interactions with other chemical substances are influenced by the modifications in the availability of its hydroxyl groups, which govern its chemistry [39,61,68]. Currently, these physical and chemical properties of cellulose at the nanoscale drive the research efforts on the isolation and production of nanocellulose fibers. These features combine to make cellulose handy for applications that depend upon hydrophilicity, chemical modifications, and the improvement of highly specific area and aspect ratios [69]. Through chemical modifications of its surface, it can be grafted with molecules and even macromolecules. Such transformations aim to improve the properties of cellulose-based nanomaterials, such as high mechanical strength and renewability [62,70,71]. Consequently, with the availability of hydroxyl groups, certain disadvantages are favored, including high moisture adsorption and low compatibility with hydrophobic (especially polymeric) matrices [72].

Nanocellulose is classified into three kinds of materials (Table 1): (*i*) cellulose nanofibrils (CNFs), also referred to as nanofibrillated cellulose (NFC); (*ii*) cellulose nanocrystals (CNCs), also referred to as nanocrystalline cellulose (NCC) and cellulose nanowhiskers (CNWs); and (*iii*) bacterial nanocellulose (BNC), also known as microbial cellulose [59,73,74]. Some authors have shown that the electrospinning technique can produce a form of nanocellulose called electrospun cellulose nanofibers (ECNFs) [75]. However, there are different approaches to obtaining pure nanocellulose, which might be separated from hemicellulose, pectin, or lignin chains from raw resources. The top-down approach explores the physical or chemical disintegration of lignocellulosic biomass, while the bottom-up approach explores the fermentation capacity of cellulose-producing bacteria. The latter aim at the fermentation of low molecular weight sugar such as D-glucose [26,69,76].

**Figure 2:**
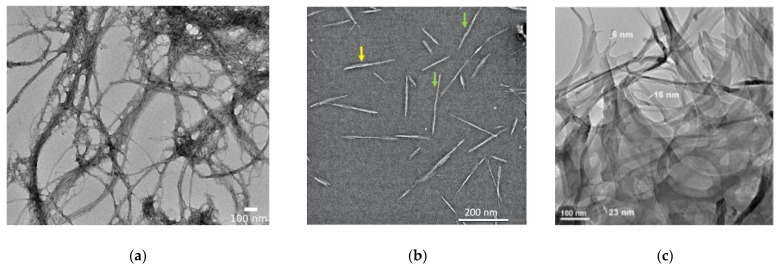
Representative micrographs for each nanocellulose material focused on in this review. Transmission electron microscopy (TEM) image of (**a**) CNFs. Reproduced (adapted) with permission [87]. Copyright 2013, American Chemical Society; (**b**) CNCs. Reproduced with permission [59]. Copyright 2020. Reproduced with permission from the American Chemical Society; (**c**) BNC [88]. Copyright 2015.

Brown [84], who observed the formation of a film during the fermentation of vinegar and referred to it as a “vinegar plant”, first described BNC in 1886. Researchers discovered that the film was constituted of cellulose and formed due to the acetic fermentation carried out by the bacterium *Acetobacter xylinum* [84]. Over the years, the genus of these bacteria has been known under several names, including *Gluconacetobacter* and currently *Komagataeibacter* [83]. When compared to plant cellulose sources, BNC has various advantageous properties, such as its higher purity (it is free of hemicellulose and lignin), higher crystallinity, good mechanical stability, smaller diameters of nanofibers (less than 100 nm versus 100 µm for typical plant cellulose bundles), and its three-dimensional nonwoven network of nanofibers [85,86]. Figure 2 shows transmission electron microscopy (TEM) graphs of cellulose nanofibrils, cellulose nanocrystals, and bacterial cellulose reported in the literature. BNC presents a structure consisting of microfibrils in the form of aggregates as ribbons (nanofibers) (Figure 2c), and more specifically as twisting ribbons. According to the literature, it has a degree of polymerization from 3000 to 9000 and a high crystallinity value (80–90%) [9].

Several methods provide functionalized BNC with various physical, rheological, mechanical, thermal, electrical, optical, and biological properties. As shown in Table 2, BNC which is pristine or combined with different components (e.g., biopolymers and nanoparticles) can be used for multiple applications, mainly because of its distinctive properties, including transparency and mechanical reinforcement.

In most cases, considering BNC’s main characteristics, a modification is suitable, as the nanomaterials based on BNC generally present high added value, with great potential for diverse applications [29]. For example, the water retention capacity of bacterial nanocellulose is almost 99% due to its hydrophilic characteristic, a feature that favors surface modifications through hydrogen bonds. The high ratio and abundance of active functional hydroxyl groups make BNC an excellent candidate for combinations with nanostructures based on inorganic and polymer nanoparticles [29]. For personalized biomedical applications, Schaffner and colleagues showed that immobilized *A. xylinum* in a 3D matrix enabled the in situ formation of BNC scaffolds. This method could demonstrate the ability to coordinate a 3D-printed nanocellulose structure in a growth medium [89]. Another study reported by Tokoh and colleagues [90] addressed the differences between BNC produced by *K. xylinus* cultivated in traditional and altered HS mediums [91]. The results revealed that the modification in the cultivation conditions altered the BNC structure, increasing the amorphous regions.

Such modifications have a crucial impact on the dissolubility and processability of BNC materials. BNC modifications can be grouped based on the functionalization condition. In situ methods add compounds during the BNC synthesis, or the bacteria growth culture, which then becomes part of the biofilm structure. Ex situ methods, such as postmodification, add compounds after the BNC has been synthesized and purified, introducing them by impregnation, loading, or coating techniques. The common compounds found on BNC composites are small biomolecules, inorganic nanoparticles, and polymers [20,29,88,92,93,94,95,96,97,98]. The advantage of bacteria-derived cellulosic microfibrils is in adjusting the culture conditions by in situ modification or modification of their structures ex situ to improve the functionality of BNC and expand its potential fields of application. Due to its structural features, BNC has shown great potential as a matrix and as a reinforcement material to synthesize various composite materials [98,99]. These approaches modify how the BNC-based nanocomposites interact with the environment and other materials [76,100,101,102,103,104]. As it is a promising material, there is a motivation to understand the different processes applied in BNC modification. It is also challenging to incorporate nanostructures to form new biocomposites capable of interacting with biological components and conducting current enzymatic biosensing devices.

**Table 2:** BNC-based nanocomposites with improved transparency and mechanical reinforcement for different applications.

	Compounds	Methods	Perspectives
Transparent composites	Chitosan [105]	Blending (casting)	Development such as transparent biodegradable and antibacterial packaging
	Poly(3-hydroxybutyrate) (PHB) [106]	In-situ polymerization	Display devices and tissue engineering scaffold
	Epoxidized Soybean Oil (ESO) [107]	In-situ polymerization	Development of “green materials” in composite material science
	Indium tin oxide (ITO)/ silicon dioxide (SiO_2_) [108]	Film Coating (sputtering and thermal evaporation)	Development of flexible organic light-emitting diodes (FOLED)
	Polycaprolactone (PCL) [109]	Blending	Preparation of fully biocompatible flexible display and biodegradable food packaging
	Poly(2-hydroxyethyl methacrylate) (PHEMA) [110]	In-situ polymerization	Development of transparent wound dressing material for skin repair
Reinforcement composites	Graphene nanosheets [111]	Blending	BNC-graphene composite films with enhanced mechanical property
	Starch [112]	Impregnation	Nanofibres were used as the biodegradable reinforcement in the starch plasticized
	Polyvinyl alcohol (PVA) [53]	In-situ impregnation	The development includes cell and tissue regeneration, controlled drug release, and the substitutes of cartilage, corneas, veins, and arteries
	Cellulose acetate butyrate (CAB) [113]	Blending (casting)	Test the hypothesis that cellulose nanocrystals obtained by acid hydrolysis from bacterial cellulose microfibrils can improve the mechanical properties of polymers
	Polypyrrole (PPy)/Ammonium Persulfate (APS) and Polypyrrole (PPy)/Iron III chloride hexahydrate (FeCl_3_·6H_2_O) [114]	In-situ polymerization	Development of an electrically conducting composite based on bacterial cellulose
	BNC/Double-walled carbon nanotubes (DWCNTs) [115]	In-situ impregnation	Flexible electrically conductive nanocomposite based on BNC cellulose and CNT
	BNC/Multiwalled carbon nanotubes (MWCNTS) [115]	In-situ impregnation	Flexible electrically conductive nanocomposite based on BNC cellulose and CNT
	BNC/poly(4-vinylaniline) (PVAN)/polyaniline (PANI) [116]	In-situ polymerization	Nanocomposites with improved functional performance, such as electrical properties.
	BNC/polypyrrole (PPy) [117]	In-situ polymerization	Production of conducting electroactive membranes from BNC coated with PPy
	BNC/graphene oxide (GO) aerogels [118]	Blending	BNC-based aerogels reinforced with GO for improved performance in different environments, envisaging lightweight structures for packaging, filters for atmosphere and water treatment, or energy applications

## 3. Bacterial Nanocellulose-Based Matrix Functionalization

There is a diversity of approaches to the improvement and modification of the capabilities of BNC materials. This type of nanocellulose can be engineered from different sources, as mentioned before, and in different shapes and different morphological forms, depending on their applications, such as hydrogels [119], membranes [120], nanofibers [121], tubes [122], spheres [123], and nanocrystals [124]. The membranes of bacterial nanocellulose are arranged in a disorderly manner, containing empty spaces, and forming a porous network. The high polarity of this material leads to its high water-holding capacity, which enables the addition of liquids (e.g., media components) and solids (e.g., polymer molecules, inorganic materials, metals, nanoparticles, etc.) as reinforcement materials. Different studies have shown that BNC can be a support or reinforcement for the formation of nano- and polymeric-composites obtained through incorporation or mixing with biocompatible, bactericidal, and electroconductive materials [35,97].

Such biomaterials may form part of excellent supports, with the immobilization of specific materials widening the applicability of BNC as a biosensing device. The fabrication of materials with specific electrical, optical, and mechanical properties [40,42,94] can be obtained by the association of organic/inorganic hybrid nanocomposites, such as plasmonic nanoparticles (NPs) [125], metal NPs [126], carbon-based nanomaterials [127], electroconductive polymers [97], and biological compounds [128]. However, in order to improve and obtain new properties for the desired application, it is necessary to (*i*) increase the electron transfer, (*ii*) immobilize these materials, and (*iii*) prevent interaction with interfering agents. In this case, refinements in the molecular composition of nanofibers are required [59,129].

**Figure 3:**
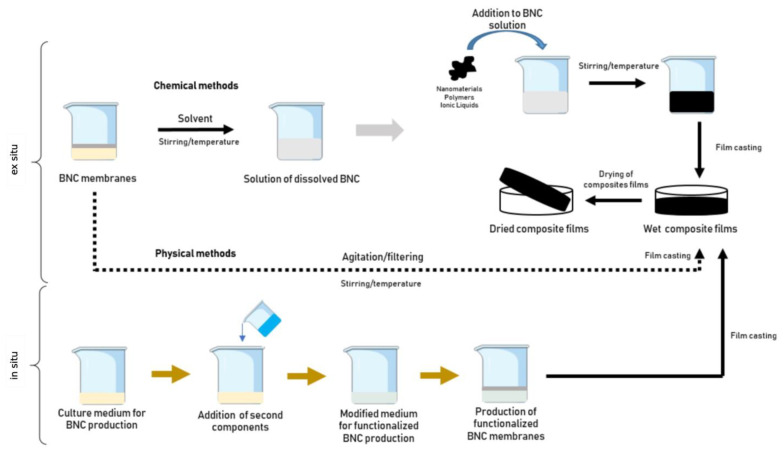
Schematic representation of BNC composites synthesized through in situ synthetic and ex situ modification strategies. The example illustrates the penetration of particles in the BNC matrix through chemical, physical, and in situ methods.

Several research groups have studied different approaches to functionalize BNC nanofibers, including in situ or ex situ approaches [92,98,130] using different forms of functionalization, such as physical, chemical [35,59,100,131], and, currently, biological modification [20,132]. In ex situ synthesis, chemical and physical methods are commonly exploited, whereas in situ synthesis can usually be formed through precipitation, sol-gel reaction [29], and, more recently, by the biological synthesis method [129]. This last method can significantly reduce the generation of the toxic residue commonly produced in chemical treatments, and it is also more selective, providing an innovative means of microbial modification that avoids the limitations of chemical and physical modification [129]. Combined with the possibility of ex situ and in situ approaches (Figure 3), these modification methods offer different possibilities according to the properties required for the application.

Torres and colleagues [133] summarized the key materials and routes used for the preparation of BNC biosensors for different types of biosensing signals to date. However, the achievement of the best approaches to and modification methods for BNC is still challenging, particularly as regards the development of electrochemical biosensors in order to obtain different sensors with specific applications. The following subsections provide an overview of the BNC functionalization methods intended to develop new biosensing strategies, as described in the literature.

### 3.1. Chemical Methods

The structure of BNC includes an abundance of hydroxyl groups, resulting in the formation of a strong hydrogen bond network and conferring to this polymer interesting features for some applications, such as a gel-like behavior and excellent mechanical properties. While its supramolecular structure influences the availability and accessibility of the hydroxyl groups, its very reactive chemistry is responsible for modifications through substitutions for other functional groups [61], utilizing grafting, for example. To introduce new functionality or charged groups into the BNC materials, chemical reagents have been used for specific substitutions of these groups, as long as they can disperse into the fibrous network and help new materials incorporate. The dispersion of these reagents occurs only in the amorphous area, which can be produced naturally or be generated by activation treatments (e.g., widening, disrupting fibrillar aggregation, troubling the crystalline order, and alkali treatment) [59,61].

As mentioned earlier, BNC presents limitations due to its rigid structure [134]. Thus, the ex situ association of nanomaterials and polymers is highly favored. The porous network structure of BNC is usually considered an advantage in most applications, however, this feature only allows submicron nanosized materials to be impregnated into the BNC matrix, limiting the possibility of associated materials. Furthermore, the uneven structure arrangements of BNC fibrils also affect the homogeneous distribution of these penetrating materials. As methods to overcome this challenge, in addition to the in situ and ex situ approaches, new synthesis routes have been investigated in the formation of BNC composites, such as synthesis from solutions of dissolved BNC [98,134]. However, the main drawback associated with this method is the limited BNC solubility. BNC is tough to dissolve due to its insolubility in water and the most widely used organic solvents. Its insolubility in nonpolar solvents is explained by its polar fibers, but its aqueous insolubility, as reported in the literature, is more challenging to comprehend [98]. One possible reason for its low solubility is the strong intra- and intermolecular bonding of BNC, which interferes with the solubilization of polar solvents (e.g., water) [98]. Only a few solvent systems or compounds have been described in the literature as capable of dissolving bacterial nanocellulose (e.g., N-methyl morpholine N-oxide (NMMO) [135]; ionic liquids [136,137]; ZnCl_2_(3H_2_O) [134]; NaOH [138]; LiOH/urea/thiourea [138,139]).

It is possible to obtain regenerated BNC composites and films from dissolved BNC by incorporating other materials in the regeneration process [98]. The introduction of conductive nanomaterials and polymers into the BNC transforms the nanofibrous structure into flexible conductive composites useful for electronic applications. Thus, functionalized fibrous nanostructures offer an efficient electrical wiring network with an active redox site through the fibers [140,141]. Chen and colleagues developed a regenerated bacterial nanocellulose/multiwalled carbon nanotube (BNC/MWCNT) composite dissolved in a dimethylacetamide/lithium chloride solvent system. The MWCNTs were wrapped in or covered by the BNC layer during the regeneration process [141].

Although BNC composites synthesized from a dissolved BNC solution are not a common approach, the mechanisms of the chemical interaction of BNC with functionalizing materials can also be improved through chemical surface modification to obtain BNC derivatives [29]. The introduction of new functional groups could enhance or add new properties such as hydrophobicity, ion adsorption capacity, mechanical, and optical properties. Thus, the chemical modifications could enable different functionalization approaches through the hydroxyl groups, improving the production of new functional BNC-based materials [35]. Some chemical methods mentioned are oxidation, esterification, etherification, amination, copolymerization, and crosslinking reactions [61].

The oxidation reaction enables the replacement of the hydroxyl groups by carbonyl and carboxyl groups. The insertion of these groups into the BNC structure can form strong covalent bonds with primary amines from biomolecules and form imine and amide bonds. The carbonyl groups can be obtained through periodate oxidation [61,68,142] or TEMPO-mediated (2,2,6,6-tetramethylpiperidine-1-oxyl) oxidation of the BNC membranes. Although they require multistep reactions to be activated and to form the covalent bonds, this approach presents a strong interaction between the substrate (cellulose) and the immobilized biomolecule [29,59,61].

Modified cellulosic materials and their derivatives, called cellulose esters and cellulose ethers, can be obtained by introducing other functional groups employing the esterification and etherification reactions, respectively. The immobilization of biomolecules on the surfaces of biosensors is often carried out on nitrocellulose (nitrated cellulose–NC) and carboxymethyl cellulose (CMC). Nitrocellulose is formed by the esterification of the hydroxyl groups from the cellulose in the presence of nitric acid (HNO_3_) under acidic conditions [61]. Although the aforementioned modifications are most often employed in plant cellulose, significant studies have been performed by subjecting BNC to esterification processes [143,144,145]. In the work of Sun and colleagues [145], nitrated bacterial cellulose (NBC) was synthesized from BNC through a sulfuric nitric acid method. They could observe from the TEM images that the bacterial cellulose nitrate presented a net structure with more holes and ribbons in disorder, in contrast to BNC. Such changes suggested that a drastic reorganization of the microfibrils of the BNC happened during the solid-phase nitration. Luo and colleagues [144] also noticed that, although NBC has the same chemical structure as NC, it possesses a high purity and a unique network structure, which shows better mechanical and safety properties than the conventional NC.

Noncovalent reactions, such as amination, which promotes chelating bonding between the biomolecule and cellulosic materials, can also be considered in the immobilization of biomolecules. The hydroxyl groups with low reactivity in the glucose chains in BNC have limited interaction with amine groups. Amino groups can directly react with the amines of enzymatic proteins or biomolecules in a reasonable approach to improving immobilization efficiency [146]. The formation of nitrilotriacetic acid (NTA) or iminodiacetic acid (IDA) matrices on cellulose membranes adds metal cations (e.g., nickel cation) to the surfaces of the fibers, which enables the immobilization of His-tagged proteins by bioaffinity attachment [61]. This specific affinity between the metal cation and a chelating agent forms a noncovalent bond, with the disadvantage of this being a reversible reaction when it is exposed to competitive agents or acid pH [61,147]. Yu and colleagues [146] used glutaraldehyde in a cross-linking reaction to form secondary amines with more stable bonds, resulting in amino-functionalized bacterial cellulose with amino-covalent bonds. This modified BNC was applied as a scaffold to immobilize horseradish peroxidase (HRP), with an improvement in heat resistance, alkali adaptability, and retention of enzymatic activity. The amine-rich scaffold could retain more than 70% of its intrinsic enzymatic activity.

Rebelo and colleagues [116] produced a modified BNC nanofibril network with an electrically conductive polyvinylanile/polyaniline (PVAN/PANI) bilayer by using a grafting method with functional group availability. Chemical grafting of BNC using polymerization is an efficacious method for surface chemical modification. This method provides lots of anchoring points for biomolecule immobilization. Copolymers may be added to enhance the functionalities on the cellulose surface. The initiation by free radical polymerization begins with the insertion of a site radical in the cellulose chains. Some polymers already have these sites or have them inserted with postpolymerization treatment [61,72]. The grafting of polymer to the BNC surface provides a versatile tool for modification and functionalization [116]. End-functionalized polymer chains may be attached to the cellulose by functional groups (grafting to), or the grafting reaction can continue by polymerization from the surface (grafting from) [148,149]. The latter is more used for the immobilization of biomolecules, in which the copolymerization chain starts at the initiation sites in the cellulose structure [61].

### 3.2. Physical Methods

BNC composites prepared from in situ synthesis modification can present some difficulties during the experimental process, such as the concentration of incorporated material, which will affect the BNC production in the culture medium [98]. However, physical methods applied during ex situ synthesis can avoid this problem [29]. In this approach, liquid substances, tiny solid particles, or NPs can easily penetrate or be deposited into the porous network of the BNC matrix. Additionally, deposited or impregnated polymers, inorganic materials, metals, and metallic oxides can promote the immobilization of biomolecules in the BNC matrix by physical forces, such as electrostatics, van der Waals, hydrophobic interactions, and hydrogen bonding [29,61,98]. Physical methods for the formation of BNC composites can be classified into physical coating (sputtering [150], thermal evaporation [108]), and impregnation (agitation [42] and vacuum-filtering [151]).

Physical coating is a widely explored approach to producing BNC membranes in substrates for the deposition of electronic arrays [108,150]. In it, BNC is used as a substrate for the deposition of organic compounds and metallic oxides in the manufacture of flexible electronic devices [108]. Modification of cellulose structures does not occur significantly by deposition, however, it produces weak interactions by hydrogen bonds between the BNC surface and the deposited material [98,132]. In this case, BNC surfaces are functionalized at a controlled temperature and concentration, keeping the three-dimensional structure almost unchanged [98]. In this study, the sputtered coating of Cu was conducted on the BNC nanofiber surface in a magnetron sputtering [150].

**Figure 4:**
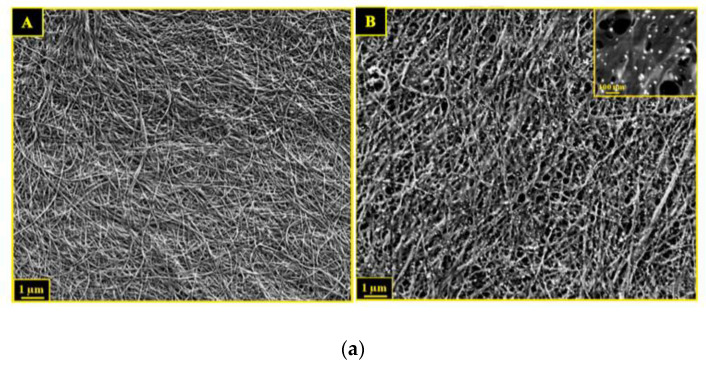

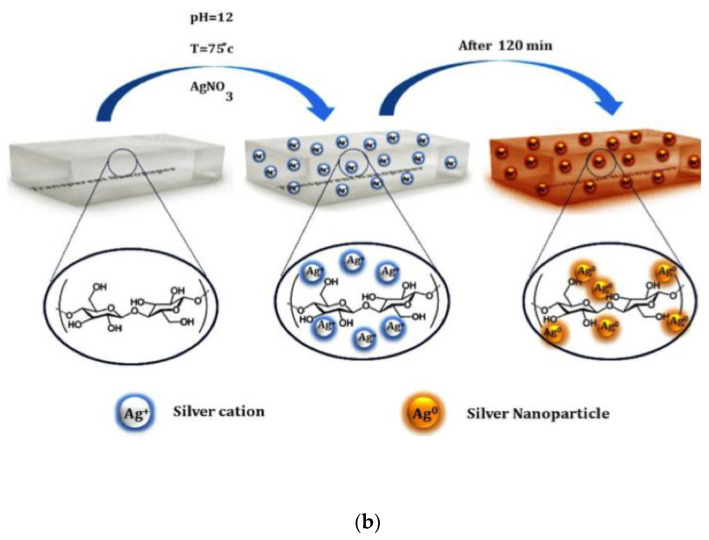
Modified BNC prepared by agitation method for incorporation of nanoparticles. (**a**) (**A**) Field Emission Scanning Electron Microscopy (FE-SEM) images of transparent nanopaper. (**B**) FE-SEM images of ESNP; (**b**) Schematic representation for the fabrication of ESNPs. Adapted with permission from [94]. Copyright 2015. Reproduced with permission from Elsevier.

On the other hand, impregnation by agitation and vacuum filtration are the most used techniques due to of their facility and availability. Through these methods, nanomaterials, ionic liquids, and polymers penetrate the nanofibers to produce BNC composites with electronic application capacity [94,152]. Although these methods have some limitations, such as the size and amount of the materials that can be incorporated, they are ecofriendly, i.e., they require fewer reagents and fewer toxic components than chemical methods [29,94]. For example, the study conducted by Pourreza and colleagues [94] showed an in situ generation of silver nanoparticles using flexible and transparent BNC nanopapers. Figure 4 presents the formation followed by the incorporation of silver nanoparticles (AgNPs) into the stirred solution to obtain a bionanocomposite called “embedded silver nanoparticles in transparent nanopaper (ESNPs).” The hydroxyl groups of the BNC matrix are capable of immobilizing the nanoparticles into the fibrous structure (Figure 4a(A)) and act as reducing agents for the formation of AgNPs (Figure 4a(B)) [94].

It is essential to highlight that physical methods, either by surface deposition or impregnation, can cause some weak physical interactions between the incorporated material and the BNC matrix [129]. However, some materials such as inorganic compounds, deposited or impregnated polymers, metals, and metallic oxides can promote the immobilization of biomolecules on BNC composites by physical forces such as hydrogen bonds, van der Waals, or electrostatic and hydrophobic interactions [29,61,98].

### 3.3. Chemical and Physical In-Situ Methods

As mentioned earlier, the BNC structure is formed by a highly porous network, and its three-dimensional nanofibers enable the diffusion of several nanomaterials during the BNC biosynthesis. Thus, in situ functionalization is one of the main differences between BNC and other cellulosic sources [98]. The reinforcement materials can be included in the BNC culture media during or at the origin of the BNC biosynthetic process.

The formation of nanocomposites based on BNC can be produced by nanoparticles doped on the nanomaterial matrix, but this matrix in particular also has great potential as a model for the controlled synthesis of nanomaterials with specific structures [29]. This process for the formation of nanomaterials can be obtained by precipitation [153], oxidation reduction [120], and sol-gel reaction [154]. In contrast, the intrinsic structure of bacterial nanocellulose can also be modified by recently developed techniques involving biological synthesis reactions. This approach has gained traction because it reduces chemical reagents in the assembly of new composites, using microbial in situ fermentation to facilitate contact between other components and the BNC matrix. It is a potential method for the biological modification of BNC that avoids the limitations of chemical and physical methods [129].

The incorporation of polymers [53], carbon-based nanomaterials [93], nanoparticles, and other materials, is used to enhance the optical and electronic characteristics. In some BNC applications, functionalization can also increase the biodegradable and biocompatible potential of the BNC-based material. However, in situ techniques present some limitations [93]. The cytotoxicity of some compounds may make BNC network formation and the metabolic activity of BNC-forming bacteria difficult. In addition, static culture synthesis is complex because the particles remain suspended in the BNC media for a short time. One possible procedure is to apply agitated culture. However, the BNC composites formed cannot be utilized in several applications [98]. Therefore, to reduce chemical reagents and improve the in situ incorporation of materials, biological synthesis reactions are currently being applied to ameliorate the intrinsic aspects or reveal new properties.

**Figure 5:**
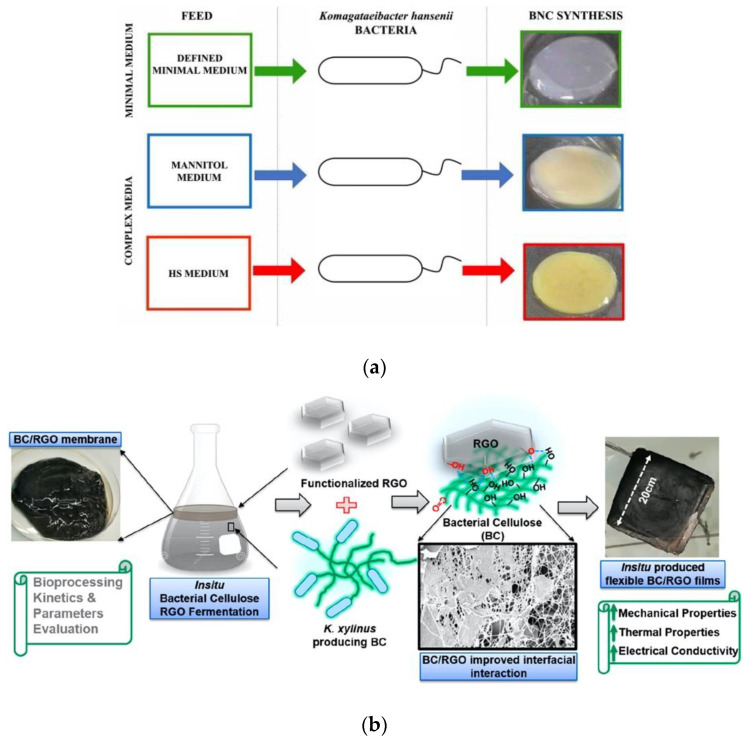

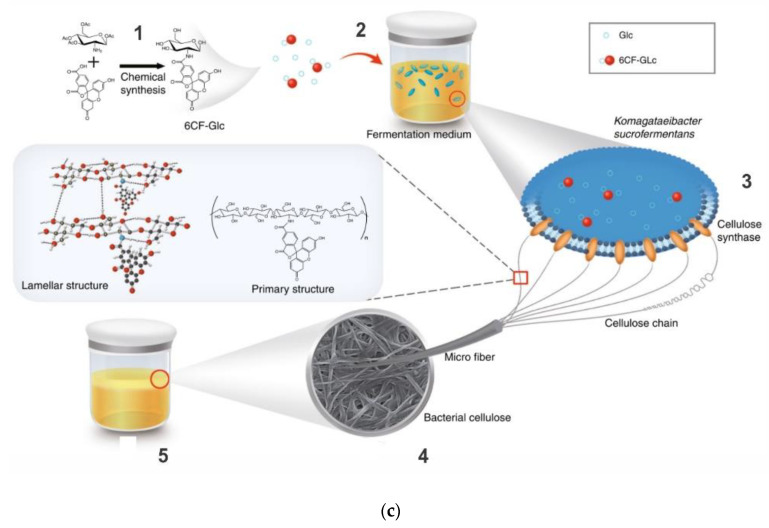
(**a**) Nanocellulose biosynthesis by *Komagataeibacter hansenii* in a defined minimal culture medium. Membranes with increased optical properties are visible in the figure. Reproduced with permission [20]. Copyright 2018. Reproduced with permission from Springer; (**b**) Schematic illustration of steps involved in the fabrication in situ of BNC/RGO nanocomposites. Reproduced with permission [93]. Copyright 2019. Reproduced with permission from the American Chemical Society; (**c**) Biosynthesis of 6CF-BNC based on an in-situ microbial fermentation method [129].

Recently, Souza and colleagues [20] reported that a modification in the composition of the culture medium for the bacteria could alter the morphological and physicochemical characteristics of BNC membranes, affecting the optical properties and the porous aspect of the membrane produced (Figure 5a). On the other hand, some approaches only involve the insertion of nanomaterials and soluble polymers into the culture medium. As shown in Figure 5b, the in situ growth or adsorption of reduced graphene oxide (RGO) on the nanocellulose fibers allows researchers to obtain conductive membranes and demonstrates the formation of a percolated network in BNC/RGO nanostructures with increased mechanical properties [93]. Other techniques that use microbial bioengineering have emerged recently. The molecular modification of glucose units labeled with 6-carboxyfluorescein (6CF) and used as a substrate for culturing the bacteria was proposed to produce a fluorescent BNC membrane (Figure 5c) [129].

## 4. Application of the Functionalized BNC in Biosensors and Future Perspectives

**Figure 6:**
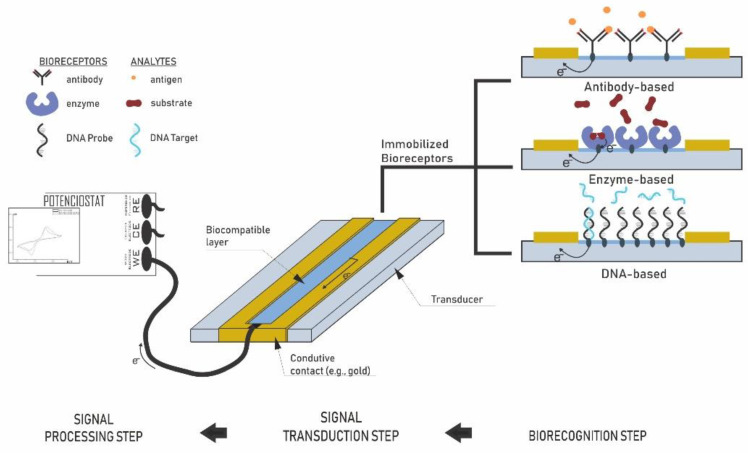
A typical design of antibody-, enzyme-, and DNA-based electrochemical biosensors, based on information from [46].

A biosensor is an analytical device capable of transforming biochemical responses into measurable signals. Its working principle is linked to three main components: a biological recognition system (bioreceptor), a physical-chemical transducer, and an electronic system that processes and displays the signal [46,155]. The first recognition interface guarantees different selectivity and accuracy according to the detection method/biological element (e.g., enzymes, antibodies, DNA, microorganisms, receptors, cells) [44,49,50,55]. The bioreceptor interacts with the target analyte, resulting in a biochemical reaction. The transducer is responsible for converting this reaction into a demonstrable signal, which is associated with the concentration of the analyte and can be quantified by phenomena based on optics, acoustics, mechanics, calorimetry, electronics, or electrochemistry [156]. These devices provide small dimensions, low-cost fabrication, and real-time detection, making them an attractive dispositive for quantitative and semiquantitative analyses [157]. Figure 6 presents the basic principle of the biosensor.

Among the different transducers available to convert biochemical signals into measurable signals, electrochemical systems have frequently been used as the detection mode in commercial biosensors. Their quantification involves detecting a redox reaction in the transducer when the bioreceptor interacts selectively with the analyte in the solution, which generates an electrochemical signal. This signal can induce amperometric and potentiometric responses, field-effect transistors (FET), and conductometric responses [52,56]. The advances in biosensing analyses require understanding the charge transport and the electron transfer that occurs in the electrode/electrolyte interface, which will lead to an efficient bond between the bioreceptor and the target analyte, and will immobilize the biomolecule in the transducer [43,47,158].

The physicochemical characteristics of the transducer interface can be adapted and improved to increase the quantitative accuracy, selectivity, and reactivity of the techniques. Constructing a modified layer is highly desirable and will allow a specific biological function to be better retained on the transducer’s surface. [128]. One approach to optimizing the electron transfer in the interface electrode/electrolyte is to design a suitable surface (support) depending on the properties and the stability of the biomolecule to guarantee its immobilization onto the electrode. Improving the biomolecule and electrode surface integration will allow a high biosensor sensitivity [159,160,161,162].

The BNC has formidable properties that make it a superb candidate to be used as support for biomolecule immobilization onto the transducer surface. Its high specific surface area and nanoporous structure, for example, facilitate the penetration of the biomolecules, resulting in higher sensitivity and faster biosensor response time [29,61,163,164]. Furthermore, this nanomaterial possesses high tensile strength, crystallinity, hydrophilicity, and great water-holding capacity [60]. However, it lacks conductivity [60,103,163,164], a drawback to its applications in electrochemical biosensing. A strategy to overcome this issue is to prepare a BNC nanocomposite that contains conductive materials, such as graphene oxide, carbon nanotubes, conductive polymers, and gold nanoparticles.

The methods of manufacturing the BNC-based materials can be carried out through the nanofabrication of materials incorporated in the BNC nanofibrils or by a polymer-based approach. BNC-based nanocomposites can have high catalytic selectivity because of their interaction with biomolecules. They can achieve dimensions between 2 and 20 nm, similar to nanoparticles (NPs), and the introduction of NPs increases the biosensors’ electronic and optical transduction characteristics [43]. The interaction of biomolecules and NPs on the polymer matrices, such as BNC, is possible by applying physical, chemical, or biological methods [43,46]. Carbon nanotubes (CNT) have also attracted interest in the manufacturing of BNC-based materials due to their high surface area, electrical conductivity, and good chemical and thermal stability. Furthermore, it has been shown that the elastic modulus and the ultimate strength of polymer composites increase even with the incorporation of small amounts of CNTs, which enhance these matrices’ mechanical and thermal properties [115].

The polymer-based approach integrates the fundamental properties of biosensing applications. Conducting polymers, such as polyaniline (PANI) [97,165] and polypyrrole (PPy) [114], have frequently been used to prepare BNC-based materials. The conjunction of the conducting characteristics of the conjugated polymers with the porous structure of nanocellulose material results in excellent sensing characteristics and elevates the BNC’s capacity to immobilize biomolecules. This happens due to BNC’s good water-holding capacity and hydrophilic properties, which enhance the conducting polymers’ physical and structural properties [98,103,163]. These polymers also enhance the transduced analytical signal generated by interacting these immobilized biomolecules with the target analyte [51,166] and, consequently, have been employed in biosensing analyses [51,98,164]. However, due to their low solubility in common organic solvents and poor mechanical properties, their application has been restricted to some electronic devices [167,168]. The following subsection demonstrates these nanocomposites’ applicability in the construction of biosensor devices.

Even though it is challenging to compare different manufacturing strategies, Table 3 presents a brief summary of the performance parameters of biosensors that contain a layout with BNC and alternative polymers (CS, Alg, and synthetic nonconductive polymers) as immobilizing substrates. The alternatives show the unique advantages and drawbacks of BNC in biosensing applications [169,170]. Nonetheless, BNC-based biosensors have performance in the same range or improved linear range and detection limits. However, when measured, their stability (preservation of functionality over time assay), repeatability (preservation of functionality over multiple tests assay), and reproducibility (preservation of functionality over the manufacture of different electrodes), present mixed positive (lower relative standard deviation (RSD)) and negative (higher RSD) results in relation to the other alternatives.

These biosensing applications do not involve any special rules or general ways to theoretically predict the biosensor performance to detect components from selecting immobilizing polymers. Nevertheless, in addition to target analytes and selected biorecognition entities, the differences in the detection achievements depend on polymer features, synergic effects through functionalization materials and methods, and performance test protocols [171,172,173,174,175]. There are a lot of combinations and facets that are yet to be explored in biosensor research with bacterial nanocellulose [175]. Given this context, the next sections are not limited to discussing BNC advantages and drawbacks but to addressing the BNC-based nanocomposites’ potential in the construction of straightforward and sustainable advanced biosensor devices.

**Table 3:** Summary comparisons between BNC-based and alternative polymer-based platforms used for biosensing applications.

Sensing Target	Immobilizing (Bio) Substrates	Sensing Platform *	Linear Range	LOD **	Stability (Loss% Per Day)	Repeatability RSD ***	Reproducibility RSD ***	Year	Ref.
H_2_O_2_	Bacterial Nanocellulose	GCE/BNC/AuNPs/HRP	N.D.	1 µM	–	–	–	2010	[104]
		GCE/BNC/AuNPs/HRP	0.3–10^3^ µM	0.1 µM	0.65%	–	–	2011	[128]
	Chitosan	GCE/LDH-cmCS/HRP	20–6 × 10^3^ µM	12.4 µM	0.98%	1.95%	2.15%	2018	[176]
	Alginate	GCE/AuNPs/L-Cys/Cell-Alg	20–100 µM	1.96 µM	1.5%	<5%	2.69–4.86%	2018	[177]
	Synthetic Polymers	GCE/Nafion/PAni-PAAm@L012	0.01–50 µM	2.9 × 10^-3^ µM	-	4.94%	–	2020	[178]
Glucose	Bacterial Nanocellulose	GCE/BNC/AuNPs/GOx-HRP	10–400 µM	2.3 µM	1.42%	1.6%	–	2010	[126]
		BNC-CNTs/GOx	–	–	–	–	–	2013	[179]
		BNC/cMWCNTs-AuNPs/GOx and Lac	0–50 × 10^3^ µM	2.87 µM	1.33%	–	–	2018	[180]
	Chitosan	AuE/CS-CAR/AuNPs/GOx	5–7 µM	5 µM	–	5%	6%	2019	[181]
	Alginate	SPGE/Ca-Alg/GOx-HRP	2 × 10^3^–12 × 10^3^ µM	126 µM	1.6%	–	–	2017	[182]
	Synthetic Polymers	FTO/PVA/nano-ZnO/GOx	0.2 × 10^3^–20 × 10^3^ µM	2 µM	0.05%	1.65%	1.21%	2020	[183]
Hydroquinone	Bacterial Nanocellulose	GCE/BNC/Nafion/AuNPs/Lac	0.03–0.1 µM	5.71 × 10^-3^ µM	0.04%	3.17%	2.65%	2016	[54]
	Chitosan	GCE/CS/GO/Lac	2–100 µM	0.26 µM	–	–	3.02%	2014	[184]
	Synthetic Polymers	AuE/PDA-Fe_3_O_4_/Lac	0.2–95 µM	30 × 10^-3^ µM	0.5%	3.2%	4.4%	2012	[185]
Microbial	Bacterial Nanocellulose	BNC/PEI/cMWCNTs/ Phage	10^0^–10^7^ CFU mL^−1^	3 CFU mL^−1^	–	9%	–	2020	[186]
		BNC/PPy/TiO_2_	0.5–4 CFU mL^−1^	0.5 CFU mL^−1^	–	–	–	2020	[187]
	Chitosan	GCE/CS/AgNPs	10–10^7^ CFU mL^−1^	248 CFU mL^−1^	–	–	–	2020	[188]
	Alginate	SPCE/Na-Alg/MWCNTs/HRP-Sfmb	10^4^–10^11^ CFU mL^−1^	3.1 × 10^3^ CFU mL^−1^	0.35%	–	7.8%	2010	[189]
	Synthetic Polymers	GCE/rGO-PVA/AuNPs/Apt	9.2–9.2 × 10^3^ CFU mL^−1^	9.34 CFU mL^−1^	–	–	–	2021	[190]
Lactate	Bacterial Nanocellulose	SPE/BNC/PBNcs/LOx	1.0 × 10^3^–24.0 × 10^3^ µM	1.31 × 10^3^ µM	–	–	–	2020	[191]
	Chitosan	SPCE/CS-Pt/Cu-MOF/LOx	0.75–10^3^ µM 4 × 10^3^–50 × 10^3^ µM	0.75 µM	~0%	–	7%	2018	[192]
	Alginate	AuE/Ca-Alg-PDDA/LOx	2–3.6 × 10^3^ µM	0.05 µM	2%	–	–	2012	[193]

* Abbreviations: BNC–Bacterial Nanocellulose; GCE–glassy carbon electrode; AuNPs–gold nanoparticles; HRP–horseradish peroxidase; LDH-cmCS – ZnAl layered double hydroxide-caroxylmethyl chitosan; L-Cys–L-Cysteine; Alg–alginate; PAni–polyaniline; PAAm–polyacrylamide; L012–8-amino-5-chloro-7-phenylpyrido [3,4-d] pyridazine-1,4(2H,3H)-dione sodium; GOx–glucose oxidase; Lac–laccase; cMWCNTs–carboxylic multi-walled carbon nanotubes; AuE–gold electrode; CS–chitosan; CAR–k-carrageenan; SPGE–screen printed gold electrode; Ca-Alg–calcium alginate; FTO–fluorinated tin oxide; PVA–polyvinyl alcohol; nano-ZnO–nanostructured zinc oxide; PDA–polydopamine; SPCE–screen printed carbon electrode; PEI–polyethyleneimine; Ppy–polypyrrole; Na-Alg–sodium alginate; Sfmb–antibodies anti-*S. flexneri*; rGO–reduced graphene oxide; Apt–aptamer; PBNcs–Prussian blue nanocubes; Pt–platinum; Cu-MOF–copper metallic framework; PDDA–poly(diallyldimethylammonium chloride). ** LOD = Limit of Detection *** RSD = Relative Standard Deviation.

### 4.1. BNC-AuNP

The application of BNC nanocomposites using gold nanoparticles (AuNP) was first described in the paper by Zhang and colleagues [104]. The glassy carbon electrode was used as the transducer and, to provide a suitable surface for the horseradish peroxidase (HRP) enzyme immobilization, the nanocomposites were applied onto its surface. For the BNC-nanocomposite synthesis, the authors used a polyethylenimine (PEI) solution that acted as a reducing agent and a linking molecule. The PEI solution was mixed with BNC nanofibers, and HAuCl_4_ was added. The effective reduction of HAuCl_4_ and, consequently, the formation of the AuNPs were demonstrated when the BNC nanofibers gradually turned purple. The formation mechanism of the nanocomposites was studied, and it was found to involve three steps. Firstly, hydrogen bonds bonded the BNC’s hydroxyl groups with the PEI amine groups. Subsequently, the free amine groups of PEI protonated and conjugated with the ion AuCl_4_^-^. Lastly, the ion AuCl_4_^-^ was reduced by PEI and nucleated on the BNC surface. To confirm that the network structure of the BNC-Au nanocomposites effectively helped entrap the biomolecule HRP and to check its biocatalytic activity, the constructed biosensor was used to determine hydrogen peroxide. For that, hydroquinone was used as an electron mediator. The electrochemical response of the biosensor with and without the BNC-Au nanocomposites was compared. For the HRP/BNC-Au/GCE biosensor, the catalytic effect of HRP was successfully observed since the hydroquinone reduction current increased significantly in the presence of H_2_O_2_. The same was not observed for the HRP/BNC/GCE and HRP/Au/GCE biosensors, which demonstrated much smaller electrocatalytic reduction peak currents toward H_2_O_2_. The results revealed that the combined effects of BNC and AuNPs, such as BNC’s biocompatibility network structure and AuNPs’ high conductivity, make the BNC-Au nanocomposites a suitable matrix for enzyme immobilization.

In order to polish up their latest work [104], Wang et al. [126] used BNC-Au nanocomposites to create a glucose biosensor. The preparation of the modified electrode included BNC-Au deposition onto the GCE surface followed by the immobilization of the catalytic enzymes glucose oxidase (GOx) and HRP. The point of using both enzymes was to minimize the interference resulting from oxidations and the reduction process of other compounds at the working potential. To investigate if the nanocomposites improved the biosensor response, the electrocatalytic activities of the enzyme toward glucose were compared in the biosensors with and without the BNC-Au. The amperometric response of the biosensor without the nanocomposites indicated that its sensitivity was not high enough to detect low concentrations of glucose. The authors attributed this result to the absence of a biocompatible environment for the enzymes, which led to the low biocatalytic activity of these biomolecules. In contrast, the biosensor containing the BNC-Au nanocomposites exhibited an excellent amperometric response even at low concentrations of glucose, confirming that, due to the BNC networking, the enzymes were effectively retained onto the transductor surface and their biocatalytic activity was preserved. Furthermore, the AuNPs increased electrical conductivity on the electrode surface, obtaining a limit of detection (LOD) of 2.3 µM.

Wang and colleagues [128] investigated whether the BNC-Au nanocomposites were suitable to immobilize proteins of different sizes, such as hemoglobin (HB) and myoglobin (MB). The transducer chosen was the glassy carbon electrode, which was modified with BNC-Au nanocomposites. The cyclic voltammetry (CV) curve of BNC-Au/GCE showed that the resistivity of Au-BNC was significantly reduced compared to BC itself, indicating that AuNPs were efficiently attached to the BNC nanostructure. Subsequently, the proteins were immobilized onto the modified electrode surface, and their biocatalytic activity was investigated by detecting H_2_O_2_ in hydroquinone (HQ) as an electron mediator. The Hb- and Mb-based biosensors presented biocatalytic activity and rapid amperometric response toward H_2_O_2_ (linear response ranged from 10 µM to 1000 µM and 10 µM to 100 µM; detection limits were 3.9 µM and 5.1 µM, respectively), proving that the BNC-Au nanocomposites were suitable to immobilize different proteins sizes.

BNC-Au nanocomposite was applied in the work of Li et al. [54] to facilitate laccase (Lac) enzyme immobilization on the surface transducer (GCE) and create a biosensor for hydroquinone detection. Therefore, vacuum filtration was applied to deposit AuNPs on the bacterial cellulose. After that, the BNC-Au was adhered to the GCE, followed by the Lac immobilization onto the transducer surface. The CV showed a satisfactory electrochemical response to the GCE/BNC-Au/Lac biosensor, which confirmed that there was direct electron transfer between the electrode surface and the electroactive center of the immobilized enzyme. The number of electroactive species present on the electrode surface was also calculated. The calculation showed a more significant concentration of electroactive species on the biosensor that used BNC along with AuNPs for the enzyme’s immobilization [194]. These results indicated that a significant amount of Lac was immobilized on the BNC’s surface, which confirmed that its substantial surface area and nanostructure help biomolecule immobilization. To finish, the response of the biosensor toward hydroquinone was assessed by direct electron transfer (DET). The immobilized enzyme showed a great biological electrocatalyst, with the linear response of the hydroquinone ranging from 30 nm to 100 nM and a detection limit of 5.71 nM.

### 4.2. BNC-Carbon Nanotubes

Kim and colleagues applied carbon nanotubes in their study [179] to promote a direct electron transfer between the biomolecule and the electrode surface. Its excellent properties, such as outstanding electrical conductivity, were mixed with the BNC’s good biocompatibility and ultrafine network to promote the enzyme’s catalytic activity. To prepare the BNC/CNT nanocomposites, the suspension of CNTs was vacuum filtered through the BNC hydrogel and, subsequently, the BNC containing the CNTs was vacuum dried. At the end of the process, a thin BNC/CNT composite film was obtained. To immobilize the biomolecule, a GOx solution was prepared in a phosphate buffer (pH 7.00) and dropped on the dried BNC/CNT film. The authors did not use supporting electrodes to measure the electrochemical performance of the BNC/CNT composite film; instead, the film itself was applied as the electrode and a silver epoxy tape was attached to the edge of the film to make an electrical contact. The CVs obtained using the BNC/CNT/GOx electrode showed peaks referring to the reduction and oxidation reaction of the redox center of GOx immobilized. This result proved there was an efficient electron transfer between redox enzymes and the BNC/CNT electrodes, and the immobilized GOx retained its catalytic ability.

Looking to develop a self-powered biosensor, Lv et al. [180] applied BNC, AuNPs, and carboxylic multiwalled carbon nanotubes (c-MWCNTs) as nanocomposites to fabricate the dispositive. A c-MWCNTs solution was first ultrasonically ground along with a BNC solution, forming a homogeneous suspension. This suspension was filtered and dried, forming a BNC/c-MWCNTs film. Subsequently, the obtained film was immersed in a solution containing PEI and HAuCl_4_ to promote AuNP formation on its surface. The carboxyl groups on the BNC/c-MWCNTs surfaces served as anchor sites for AuNP nucleation, an interaction that prevented aggregation of AuNPs during reduction. For the fabrication of the self-powered biosensor, the researchers applied two enzymes: glucose oxidase (GOx) acting as bioanode and a Lac-based biocathode, wherein both immobilized onto the BNC/c-MWCNTs/AuNPs solution by electrostatic attraction. The electrochemical behavior of both bioanode and biocathode was investigated by CV. GOx-modified BNC/c-MWCNTs/AuNPs exhibited a pair of redox peaks that were attributed to the redox reaction of the GOx immobilized. In the same way, Lac-modified BNC/c-MWCNTs/AuNPs were investigated and the biocathode exhibited a pair of well-defined reduction and oxidation peaks. These results confirmed that there was direct electron transfer between the electrode surface and the electroactive center of the enzymes, implying that the enzyme’s electron transfer could be conducted through AuNPs and c-MWCNTs on the BNC. This further indicated a good coupling between the enzymes and BNC/c-MWCNTs/AuNPs electrodes, which was associated with the BNC’s nanofiber network and biocompatibility.

### 4.3. BNC-Conductive Polymers

Developing an electrochemical biosensor for bacterial detection requires an adequate substrate for the bacteriophages’ immobilization. In addition to being biocompatible and having a surface area that allows the immobilization of the phage particles, the bioprobe demands an ambient that preserves its tail’s ability to infect the host bacterium. As BNC can meet such requirements and offer a nontoxic environment, Farooq and colleagues [186] applied it to create a biosensor for detecting *S. aureus*. As BNC lacks conductivity, carboxylated multiwalled carbon nanotubes (c-MWCNTs) were attached to the BNC matrix to impart electrical conductivity. Phase immobilization is frequently done by the electrostatic approach, which requires an interaction between the phage capsid proteins, which have a negative charge, and the substrate. Here, PEI was added to provide a positive charge on the surface of BNC/c-MWCNTs nanocomposites. DPV analyzed the biosensor electrochemical response, and the results showed a current increase along with the bacteria concentration, which determined the *S. aureus* density. These results validated the hypothesis that the BNC nanocomposites are a suitable environment for bacteriophage immobilization.

Polypyrrole is a highly conductive polymer that enhances the electrochemical response in sensing analyses. Ghasemi and colleagues [187] demonstrated the use of this polymer associated with the BNC nanostructure and TiO_2_-Ag nanoparticles to monitor the growth of pathogenic bacteria in food. The BNC/PPy/TiO_2_-Ag nanocomposite was synthesized by chemical polymerization. In this approach, the BNC film is the transducer itself, and there is no need for a support electrode. The sensor was connected to a multimeter, and the film’s resistance change was measured. Both gram-positive and gram-negative bacteria were used to evaluate the sensor’s response to the bacteria. Centrifuged suspensions of the bacteria were added to the film in different concentrations, and the relative electrical resistance difference (RRD) was recorded. The researchers also investigated the sensor’s response by applying different PPy concentrations to the BNC nanocomposite’s fabrication. The results showed that the sensor’s sensitivity was increased by increasing the amount of PPy until it reached a maximum value that started lowering the sensitivity. These data showed the value of applying the right amount of conductive polymers and demonstrated how it will help to achieve sensitive electrodes.

### 4.4. Future Perspectives

The increasing use of nanocellulose in recently published articles on biosensors shows tremendous results on a laboratory scale [16,195]. However, successful materials for biosensing commercial solutions present a main challenge: industrial scale [16]. In previous sections, we emphasized the BNC potential for several electrochemical biosensing applications. Bacterial nanocellulose fits the new paradigm for sensing applications that consist of sustainable and robust frameworks [16]. This nanocellulose resource joins the growing field of cellulose in bioelectronics showing modular modifications during its production and suitable properties for advanced nanoscale composites, such as for flexible and miniaturized devices.

To ensure cost effectiveness, the BNC production scale up can be optimized in different steps from feedstock to functionalization [195]. As demonstrated by Abol-Fotouh and colleagues, expensive substrates for a bacteria culture medium might be replaced by renewable feedstocks such as sugar cane bagasse, wood processing residues, or agro-industrial waste [30]. Beyond bioprocess technologies, straightforward techniques have been developed from advances in bioengineered and synthetic biology studies, offering new strategies related to biosynthetic and genetic modifications through the cellulose synthesis pathway [195,196]. These genetic approaches have been showing promising results in increased cellulose production [197], as well as in rationalizing the BNC functionalization design in molecular [129] and 3D levels [198].

In the next few years, bacterial nanocellulose and its synthesized machinery might be engineered at the DNA level to achieve in vivo functionalization, discarding the need for chemical and physical functionalization steps [195]. Indeed, synthetic biology approaches have already exploited fibrous amyloid protein polymer production, which might suffer modifications through rational engineering with new protein modules and bacterial cellulose fibers [199,200]. Although advances on the genetic scale are still in the early stages, Gao and colleagues [129] demonstrated that modifications on sugar substrate could turn into BNC with new properties and morphology without any modification in the bacterial fermentation pathway.

In addition to genetic modifications of nanocellulose synthesis machinery, BNC has been applied as a sustainable and modulated scaffold for engineered living material, in which an engineered living cell could be embedded into a nanocellulose membrane (or another biomaterial) or cocultivated with a nanocellulose-producing bacteria. These materials are dynamic and responsive, with programmable properties, and might play diverse roles in wound healing, tissue engineering, antibacterial treatment, or biosensing [198,201]. In a proof of concept, Long and colleagues [202]. built a cell-based sensor platform to test the ability of nanocellulose to preserve cell viability and bioactivity in a highly efficient adhesion strategy. An engineered *Escherichia coli* with a recombinant surface-exposed CBM2a (Carbohydrate-binding module-2a) and an L-arabinose biosensing genetic system was embedded into BNC carriers. The bacteria bound tightly to BNC carriers without any substrate modification and could optically report the presence of L-arabinose in water and soil samples. One year earlier, a similar approach had been taken by Farooq and colleagues [186] without genetic modification with phages for pathogen detection.

Furthermore, the increased interest in wearable electronic devices has required flexible biosensors [203]. The substrate for fabricating this wearable sensing platform requires improved mechanical flexibility, chemical and thermal stability, biocompatibility, and conformal contact with the skin [191,204,205,206]. Paper-based biosensors, such as dipstick, lateral flow assay, and microfluidic paper-based analytical devices, have received significant attention as they allow for low-cost, portable, and disposable platforms. Nanopaper that is made entirely of BNC has all the advantageous features exhibited by conventional paper, such as versatility, abundance, transparency, flexibility, and cost. Nanopaper also obviates the drawbacks mentioned earlier by offering much lower thermal expansion and much higher chemical, mechanical, and thermal stability [40].

In the study by Naghdi and colleagues [207], the team explored optical transparency, high flexibility, porosity, biodegradability, and printability to develop a BNC-based optical sensor. The device aimed at the visual sensing of human serum albumin (HSA) in human blood serums via curcumin embedded in bacterial cellulose nanopaper. The authors developed a “lab-on-nanopaper” device that was entirely ecofriendly owing to their use of curcumin and nanopaper as safe, nontoxic, and containing green materials, with a lack of need for sophisticated instrumentation and using the minimum required sample volume (~5 µL) for HSA detection.

Gomes and colleagues [191] developed an electrochemical biosensor made on BNC substrate for lactate detection in artificial sweat. The strategy of enzymatic immobilization was based on the direct covalent binding of biomolecules with the functionalized bacterial cellulose substrate instead of immobilization onto the electrode surface. The mechanical tests showed that BNC had a remarkable capacity to stretch and could be used as a substrate in wearable devices. Although bacterial and vegetal cellulose have similar chemical compositions, BNC possesses a greater surface area and exhibits superior mechanical properties. When comparing it with previously reported wearable lactate biosensors, they found that the researchers’ proposed sensor exhibited a similar linear range, and their approach offered significant advantages regarding fabrication strategies. Furthermore, they proposed a biocompatible substrate and superior flexible properties.

## 5. Conclusions

This review provided an overview of the primary and applied concepts of nanosized cellulose, including the synthesis, characterization, and properties of nanocellulose-based materials. The specific topic highlighted herein was bacterial nanocellulose, due to its importance to nanotechnology and an extensive range of applications. BNC-based composites have attracted increased interest due to their inherent properties, which can be modulated by chemical, physical, and biological methods for BNC functionalization. In this way, the anchorage of biomolecules and assembly of third-generation biosensors might benefit from the advantages and properties of BNC-based composites. The blossoming of biosensors with a wide dynamic range, good stability, elevated reproducibility, soaring sensitivity, and fast electron transfer will offer valuable tools to be applied in medicine, environmental monitoring, food quality control, and other fields. Thus, biosensor researchers are taking advantage of novel and smart materials to simplify this technology and improve the sensors’ overall performance and main characteristics such as sensitivity, specificity, and reproducibility. In this context, BNC-based composites present critical characteristics that will assist in developing bioelectronic devices, especially biosensing devices. With the increasing number of techniques exploring the potential of bacterial nanocellulose as a biomaterial, we expect that many of the biosensors that are currently based on plastic, glass, or paper platforms will be fabricated based on BNC platforms. In the mid/long term, BNC will revolutionize state-of-the-art biosensing technology.

## Figures and Tables

**Table 1:** Classes of nanocellulose type, production methods, sources, and its average dimensions.

Nanocellulose	Method of Production	Typical Sources	Average Dimensions
Cellulose nanofibrils (CNF) [75,77,78]	High-pressure homogenization and/or grinding	Wood, cotton, tunicate, bamboo	Diameter: 2–60 nm Length: a few microns (depending on the cellulose source)
Cellulose nanocrystals (CNC) [79,80]	Acid hydrolysis	Wood, cotton, potato, flax	Diameter: 5–30 nm Length: 100–500 nm (plant cellulose)
Bacterial nanocellulose (BNC) [81,82,83]	Biosynthesis of carbon source	*Komagataeibacter* species	Diameter: 10–100 nm Length: up to 10 µm

## Data Availability

Not applicable.
